# Were Ancestral Proteins Less Specific?

**DOI:** 10.1093/molbev/msab019

**Published:** 2021-02-02

**Authors:** Lucas C Wheeler, Michael J Harms

**Affiliations:** 1 Institute of Molecular Biology, University of Oregon, Eugene, OR, USA; 2 Department of Chemistry and Biochemistry, University of Oregon, Eugene, OR, USA; 3 Department of Ecology and Evolutionary Biology, University of Colorado, Boulder, CO, USA

**Keywords:** ancestral sequence reconstruction, specificity, protein evolution, S100, phage display, phylogenetics

## Abstract

Some have hypothesized that ancestral proteins were, on average, less specific than their descendants. If true, this would provide a universal axis along which to organize protein evolution and suggests that reconstructed ancestral proteins may be uniquely powerful tools for protein engineering. Ancestral sequence reconstruction studies are one line of evidence used to support this hypothesis. Previously, we performed such a study, investigating the evolution of peptide-binding specificity for the paralogs S100A5 and S100A6. The modern proteins appeared more specific than their last common ancestor (ancA5/A6), as each paralog bound a subset of the peptides bound by ancA5/A6. In this study, we revisit this transition, using quantitative phage display to measure the interactions of 30,533 random peptides with human S100A5, S100A6, and ancA5/A6. This unbiased screen reveals a different picture. While S100A5 and S100A6 do indeed bind to a subset of the peptides recognized by ancA5/A6, they also acquired new peptide partners outside of the set recognized by ancA5/A6. Our previous work showed that ancA5/A6 had lower specificity than its descendants when measured against biological targets; our new work shows that ancA5/A6 has similar specificity to the modern proteins when measured against a random set of peptide targets. This demonstrates that altered biological specificity does not necessarily indicate altered intrinsic specificity, and sounds a cautionary note for using ancestral reconstruction studies with biological targets as a means to infer global evolutionary trends in specificity.

## Introduction

Changes in protein specificity are essential for evolution (Carroll et al. 2008; Khersonsky and Tawfik 2010; Soskine and Tawfik 2010; Kanzaki et al. 2012; Reinke et al. 2013; Kaltenbach and Tokuriki 2014; Clifton and Jackson 2016; Alhindi et al. 2017). One intriguing suggestion is that, on average, proteins become more specific over evolutionary time (Jensen 1976; Khersonsky and Tawfik 2010; Copley 2012; Wheeler et al. 2016). If true, this would be a directional “arrow” for protein evolution (Gaucher et al. 2008; Mannige et al. 2012; Risso et al. 2014; Wheeler et al. 2016). Such proposed trends are controversial (Williams et al. 2006; Wheeler et al. 2016), but could ultimately provide fundamental insights into the evolutionary process. For example, increasing specificity might indicate that proteins become less evolvable over time, as they have fewer promiscuous interactions that can be exploited to acquire new functions (Khersonsky and Tawfik 2010; Copley 2012). From a practical standpoint, it has also been suggested that less-specific reconstructed ancestors would be powerful starting points for engineering new protein functions (Risso et al. 2013).

There are several reasons that proteins may, on average, evolve towards higher specificity. First, gene duplication followed by subfunctionalization could lead to a partitioning of ancestral binding partners between descendants, and thus increase specificity along each lineage (Hittinger and Carroll 2007; Eick et al. 2012; Clifton and Jackson 2016; Alhindi et al. 2017). Second, as metabolic pathways and interaction networks become more complex, proteins must use more sophisticated rules to “parse” the environment: if an ancestral protein had to discriminate between fewer targets than modern proteins, it could be less specific and still achieve the same biological activity (Eick et al. 2012). Finally, on the deepest evolutionary timescales, it has been pointed out that the proteome of the last universal common ancestor was small. As a result, each protein would have been required to perform multiple tasks and hence have lower specificity (Jensen 1976; Copley 2012).

**Fig. 1.**
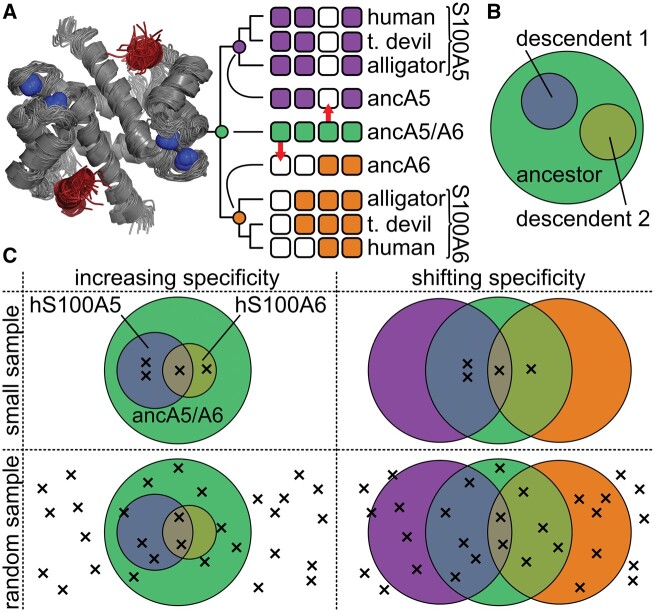
Testing the increased specificity hypothesis requires an unbiased sampling of targets. (*A*) Experimentally measured changes in peptide binding specificity for S100A5 and S100A6 (taken from Wheeler et al. 2018). Structure: location of peptide (red) binding to a model of S100A5 (gray, PDB: 2KAY). Bound ${\text{Ca}}^{2+}$ are shown as blue spheres. Phylogeny: Boxes represent the binding of four different peptides (arranged left to right) to nine different proteins (arranged top to bottom). A white box indicates the peptide does not bind that protein; a colored box indicates the peptide binds. Colors denote ancA5/A6 (green), S100A5 (purple), and S100A6 (orange). Red arrows highlight ancestral peptides lost in the modern proteins. (*B*) Venn diagram of the increasing specificity hypothesis. The large circle is the set of targets recognized by the ancestor; the smaller circles are sets of targets represented its descendants. (*C*) Venn diagrams show overlap in peptide binding sets between ancA5/A6, S100A5, and S100A6. Crosses denote experimental observations. Columns show two evolutionary scenarios: increasing specificity (left) versus shifting specificity (right). Rows show two different sampling methods: small sample (top) versus random sampling (bottom). Colors are as in (*B*).

Much of the empirical support for the increasing specificity hypothesis comes from ancestral reconstruction studies (Carroll et al. 2008; Eick et al. 2012; Risso et al. 2013, 2014; Pougach et al. 2014; Zou et al. 2015; Clifton and Jackson 2016; Devamani et al. 2016; Ma et al. 2016; Rauwerdink et al. 2016; Alhindi et al. 2017; Wheeler et al. 2018). The results from one such study are shown schematically in figure 1*A*. We previously studied the evolution of peptide binding specificity in the amniote ${\text{Ca}}^{2+}$-binding proteins S100A5 and S100A6 (Wheeler et al. 2018). Upon ${\text{Ca}}^{2+}$ binding, these proteins interact with ≈12 amino acid linear peptide regions of target proteins to modulate their activity (fig. 1*A*). S100A5 and S100A6 play regulatory roles in processes such as olfactory signaling and cell migration (Santamaria-Kisiel et al. 2006; Lee et al. 2008; Bertini et al. 2009; Leclerc et al. 2009; Słomnicki et al. 2009; Streicher et al. 2009; Liriano 2012; Donato et al. 2013). These proteins bind to peptides with diverse sequences with *K*_D_ values in the μM range (Lee et al. 2008; Streicher et al. 2009; Wheeler et al. 2018). In our previous evolutionary study, we found that S100A5 and S100A6 orthologs bound to distinct peptides, but that the last common ancestor bound to all of the peptides we tested (fig. 1*A*) (Wheeler et al. 2018). Other studies, probing other classes of interaction partners, have found similar results: the ancestor interacts with a broader range of partners than extant descendants (Carroll et al. 2008; Eick et al. 2012; Risso et al. 2013, 2014; Pougach et al. 2014; Zou et al. 2015; Clifton and Jackson 2016; Devamani et al. 2016; Ma et al. 2016; Rauwerdink et al. 2016; Alhindi et al. 2017; Wheeler et al. 2018).

But do such experiments truly test the increasing specificity hypothesis? The hypothesis can be represented as a Venn diagram: the set of targets recognized by the ancestor is larger than the sets of targets recognized by its descendants (fig. 1*B*). Results such as those in figure 1*A* are not, however, sufficient to resolve this Venn diagram. Figure 1*C* illustrates two radically different Venn diagrams consistent with our experimental observations of peptide binding in figure 1*A*. One possibility is increasing specificity (the descendant sets are smaller than the ancestral set). Another possibility is shifting specificity (the descendant sets remain the same size but diverge in their composition). Testing only a small or biased set of binding partners could lead to incorrect conclusions about the evolutionary process. Distinguishing the possibilities shown in figure 1*C* requires estimating the populations in each region of the Venn diagram, which can only be done with a much larger, unbiased sample of the set of binding partners.

To test for the evolution of increased specificity, we set out to estimate changes in the total set of peptides between ancA5/A6 and two of its descendants–human S100A5 (hA5) and human S100A6 (hA6). This evolutionary transition is an ideal model to probe this question. We already have a reconstructed ancestral protein that exhibits an apparent gain in specificity over time for both proteins, at least for a small collection of peptides (Wheeler et al. 2018). Further, because they bind to ≈12 amino acid peptides, the set of binders is discrete and enumerable ($20^{12}=4\times 10^{15}$ targets). This contrasts with interactions between proteins and small molecules, for which there is an effectively infinite chemical “space” of possible moieties to sample. Although we cannot measure the entire space of 12-mer peptides, its discrete nature means we can learn about the average features of the space by randomly sampling amino acids at each site in the 12-mer peptides.

We set out to estimate changes in the total sets of partners recognized by these proteins using a high-throughput characterization of peptide binding. We found that the modern proteins bound to a similar number of targets as the ancestor, and that both hA5 and hA6 acquired a large number of new targets since ancA5/A6. Thus, the original observation that a smaller number of targets are bound by the ancestor relative to the modern proteins reflects a shifting set of targets—not a shrinking set. This suggests that the evidence for a global trend towards increased specificity from less-specific ancestral states should be revisited.

## Results

### Peptide/Protein Interactions Measured by Phage Display

Our goal was to measure changes in the total binding sets between human S100A5 (hA5), human S100A6 (hA6), and their last common ancestor (ancA5/A6) (Wheeler et al. 2018). To account for uncertainty in the reconstruction, we also characterized an alternate reconstruction of ancA5/A6 (altAll) that incorporates alternate amino acids at uncertain positions in the reconstruction (Eick et al. 2017). The altAll sequence is an aggressive attempt to incorporate uncertainty, as it simultaneously flips all uncertain sites to their next-most-probable amino acid state in one sequence. (This protein differs at 21 of 86 sites from ancA5/A6). Because of this procedure, the altAll ancestor likely has more errors in its reconstruction than ancA5/A6 (Eick et al. 2017). Despite this, the altAll ancestor behaved similarly to ancA5/A6 in our previous experiments (Wheeler et al. 2018).

We wanted to study the interaction of tens of thousands of random peptides to each protein using phage display (Sidhu et al. 2000; Fowler and Fields 2014). As input for the screen, we selected a commercial library of randomized 12-mer peptides expressed as fusions with the M13 phage coat protein pIII. Each phage particle has five identical peptides on its surface. These peptides have a free N-terminus followed by a shared six amino acid C-terminus that links them to the pIII protein. The sequence of each peptide is thus XXXXXXXXXXXXGGGSAE, were “X” is a randomized position. We showed previously that the GGGSAE flank has no effect on S100 peptide binding (Wheeler et al. 2018).

**Fig. 2.**
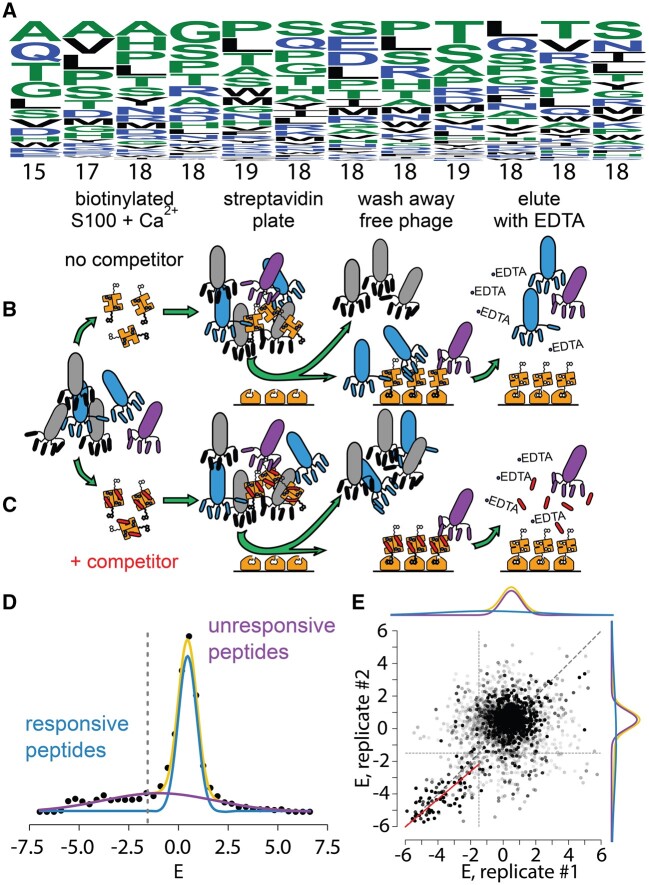
Set of binding peptides can be estimated using phage display. (*A*) Amino acids were observed at each of the 12 positions in the random peptide library prior to the experiment. The height of each letter corresponds to its frequency. Amino acids are sorted from most common to least common, top to bottom. The number of amino acids at that position with a frequency above 1% is shown below each column. (*B*, *C*) Rows show two different experiments, done in parallel, for each protein. Biotinylated, ${\text{Ca}}^{2+}$-loaded, S100 is added to a population of phage either alone (row B) or with saturating competitor peptide added in trans (row B). Phage that binds to the protein (blue or purple) are pulled down using a streptavidin plate. Bound phage is then eluted using EDTA, which disrupts the peptide binding interface. In the absence of competitor (row B), phage bind adventitiously (purple) as well as at the interface of interest (blue). In the presence of a competitor (row C), only adventitious binders are present. (*D*) Distribution of enrichment values for peptides taken from pooled biological replicates of hA5. The measured distribution (black points) can be fit by the sum of two Gaussian distributions: responsive (blue) and unresponsive (purple), which sum to the total (yellow). The dashed line indicates cutoff for E≤−1.5 used throughout the manuscript. (*E*) The responsive distribution for hA5 is correlated between biological replicates, while the unresponsive distribution is not. Axes are an enrichment for replicate #1 or replicate #2. Points are individual peptides seen in both replicates. Distributions for each replicate are shown on the top and right, respectively. The red dashed line is the best fit line (orthogonal distance regression) between the replicates for the E≤−1.5 region ($R^{2}=0.70$).

Prior to our experiments, we sequenced the naive library to assess its composition. Figure 2*A* shows the relative frequency in the library of each of the 20 amino acids at all 12 positions in the peptide. All positions have many amino acids represented at high frequency. To quantify the diversity, we calculated the number of amino acids with a frequency above 1% at each position (shown below each position in fig. 2*A*). Ten of the 12 sites have 18 or 19 amino acids represented; the least diverse sites still have 15 and 17 amino acids, respectively. This library thus represents an approximately random sampling of the peptide sequence space of the sort described in figure 1*C*.

We next set out to measure the binding of S100 proteins to this library of random 12-mer peptides. The S100 peptide-binding interface is only exposed upon ${\text{Ca}}^{2+}$-binding (fig. 1*A*); therefore, we performed phage panning experiments in the presence of saturating ${\text{Ca}}^{2+}$ and then eluted the bound phage using saturating ethylenediaminetetraacetic acid (EDTA) (fig. 2*B*). After this panning step, the population of enriched phage particles will be a mixture of those that bind at the site of interest and those that bind adventitiously (blue and purple phage, fig. 2*B*) (Sidhu et al. 2000; Willats 2002). To separate these populations, we repeated the panning experiment in the presence of a saturating concentration of competitor peptide known to bind at the site of interest (GFDWRWGMEALTGGGSAE, fig. 2*C*) (Wheeler et al. 2018). This should lower the enrichment of peptides that bind at the site of interest, while allowing any adventitious interactions to remain. By comparing the competitor and noncompetitor pools, we can distinguish between specific and adventitious binders.

We performed this experiment with and without a competitor, in biological duplicate, for all proteins. We found that phage panned in the presence of 2 mM ${\text{Ca}}^{2+}$ and eluted with 5 mM EDTA phage enriched strongly for all proteins relative to a biotin-only control (supplementary fig. S1, Supplementary Material online). Further, the addition of competitor binding knocked down enrichment in all samples (supplementary fig. S1, Supplementary Material online). We showed previously that enrichment is strictly dependent on ${\text{Ca}}^{2+}$: we see no phage recovery above our biotin-only controls if we pan with S100 proteins in the presence of 2 mM EDTA and elute with 5 mM EDTA (see supplementary fig. S1, Supplementary Material online, in Wheeler et al. 2018).

After panning, we sequenced the resulting phage pools using Illumina sequencing. We applied strict quality control, discarding any peptide that exhibited less than six counts (see Methods, supplementary fig. S2, Supplementary Material online). After quality control, we had a total of 265 million reads spread over 17 samples (supplementary Table S1, Supplementary Material online). We estimated changes in the frequencies of peptides between samples with and without competitor peptide (supplementary fig. S3, Supplementary Material online). For each peptide, we determined *E*:

(1) $$E=-\text{ln}\left(\frac{c_{\text{non}-\text{competitor}}}{c_{\text{competitor}}}\right)$$

where *c*_competitor_ and $c_{\text{non}-\text{competitor}}$ are the sequence counts for the peptide recovered from parallel ${\text{Ca}}^{2+}/\text{EDTA}$ panning experiments done with and without competitor peptides.

If *E *<* *0, the competitor peptide interferes with the ${\text{Ca}}^{2+}$-dependent enrichment of the peptide. The simplest interpretation of this result is that the phage peptide binds at the canonical S100 peptide interface (fig. 1*A*), and is thus disrupted with the addition of competitor. If *E *=* *0, the addition of competitor has no effect on panning, meaning the phage peptide either did not bind or bound at a site away from the canonical peptide binding site. If *E *>* *0, the competitor peptide improves ${\text{Ca}}^{2+}$-dependent enrichment of the phage peptide. This could occur if the phage peptide binds more favorably to the S100/competitor peptide complex than the S100 alone. Such enrichment would still be ${\text{Ca}}^{2+}$-dependent because binding of the competitor peptide is itself dependent on ${\text{Ca}}^{2+}$ (Wheeler et al. 2018): addition of EDTA disrupts the S100/competitor complex, and thus, elutes any phage peptides that bind preferentially to the S100/competitor complex (fig. 2*C*).

We determined *E* for hA5, hA6, ancA5/A6, and altAll. We found that the distribution of *E* for each protein could be described using two Gaussian distributions, apparently reflecting two underlying processes (fig. 2*C*, supplementary fig. S4, Supplementary Material online). The dominant peak, centered about *E *=* *0, consists of “unresponsive” peptides whose frequencies change little in response to competitor peptide. A second, broader, distribution describes “responsive” peptides whose frequencies change with the addition of competitor. The responsive distribution was shifted towards negative values, meaning the addition of competitor generally disrupts phage peptide binding. There were, however, responsive peptides with *E *>* *0, meaning that competitor peptide enhanced enrichment. As described above, such peptides are not of interest in this study; therefore, we focused our efforts on the *E *<* *0 portion of the responsive distribution.

We sought to identify a cutoff in *E* that would allow us to isolate and study the *E *<* *0 region of the responsive distribution corresponding to specifically enriching peptides. We used the mean and standard deviations of the responsive and unresponsive distributions to calculate their probability density as a function of *E*: ${\text{pdf}}_{\text{responsive}}(E)$ and ${\text{pdf}}_{\text{unresponsive}}(E)$. We then calculated the posterior probability that a peptide with a given value of *E* arose from the unresponsive distribution by $p_{\text{posterior}}={\text{pdf}}_{\text{unresponsive}}(E)/({\text{pdf}}_{\text{responsive}}(E)+{\text{pdf}}_{\text{unresponsive}}(E))$. We found that *p*_posterior_ = 0.05 occurred around E=−1.5 for all proteins (fig. 2*C*, supplementary figs. S4 and S5, Supplementary Material online). We, therefore, interpret peptides with E≤−1.5 as unambiguously arising from the responsive distribution. These are the peptides of interest for this study.

If the responsive distribution reflects specific binding and the unresponsive distribution reflects nonspecific binding, we would predict a high correlation between biological replicates for the responsive distribution and a weak correlation for the unresponsive distributions. To test this, we plotted the *E* distributions for the biological replicates of hA5 against one another (fig. 2*E*). As predicted, we see a strong correlation between replicates for the responsive distribution (E≤−1.5; $R^{2}=0.70$). In contrast, the unresponsive distributions are uncorrelated between replicates, yielding a cloud of points for E>−1.5. We are thus confident that the unresponsive distribution reflects nonspecific changes in phage frequency and should be ignored in downstream analyses.

### Peptide/Protein Interactions Measured by Fluorescence Polarization

We next set out to determine the relationship between enrichment and peptide-binding affinity. We selected nine peptides for which we had estimates of *E* for hA5, hA6, and ancA5/A6 from our phage display experiments. The peptides had *E* values ranging from –6 to 6. Some peptides enriched universally: EGLDLMSILELIGGSAE, for example, had E≈−4 for all three proteins. Other peptides are enriched for only a specific protein. For example, SRQTTSTHEWVVGGSAE had *E* = – 6 for hA6 and E=−0.7 and –0.4 for hA5 and ancA5/A6, respectively. The full set of peptides, with their enrichment values, is given in supplementary table S2, Supplementary Material online.

**Fig. 3.**
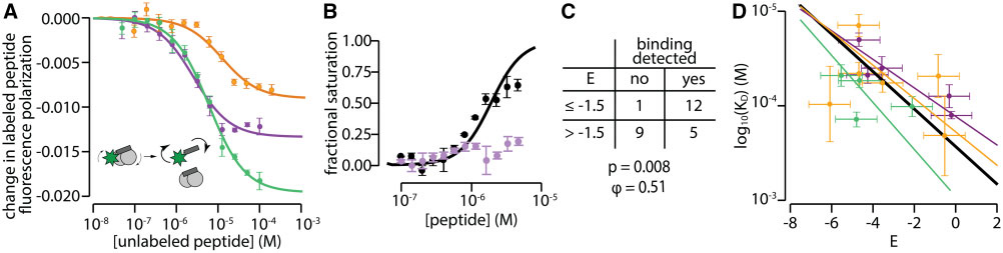
Peptide enrichment indicates peptide binding. (*A*) Unlabeled competitor peptide competing off the fluorescently labeled probe peptide for hA5 (purple), hA6 (orange), and ancA5/A6 (green). The points are experimentally measured data points (single biological replicate; error bars are standard deviation of technical replicates; lines are a binding model fit to the data. The inset graphic shows the experimental design. In the absence of competitor, polarization is high because the probe peptide is bound to the protein and thus rotates slowly. In contrast, once a competitor peptide is added, the probe is displaced, leading to faster rotation and a loss of probe signal. (*B*) Binding curves for two representative peptides binding to hA5 as measured by displacement of the fluorescently labeled probe: black (EGLDLMSILELIGGSAE; E=−4.3) and pink (SRQTTSTHEWVVGGSAE; E=−0.7). No model could be reliably fit to the pink points. (*C*) Contingency table relating *E* to binding. Entries in the table correspond to protein/peptide pairs in the set of 27 (hA5, hA6, and ancA5/A6 binding to nine different phage peptides). The *χ*_2_*P* value and Matthews coefficient for the table are show below, indicating there is a statistically significant, positive relationship between *E* class and the ability to detect binding. (*D*) Quantitative correlation between $log_{10}(K_{D})$ and *E* for each peptide. Lines were determined by orthogonal distance regression. The colors denote different proteins: hA5 (purple), hA6 (orange), and ancA5/A6 (green). The solid black line denotes a fit to all 17 points.

To mimic the phage display experiment, we measured binding by displacement of a fluorescently labeled probe peptide. For our probe, we used the competitor peptide from the phage display experiment with the addition of an N-terminal 5-FAM fluorophore (fig. 3*A*). We validated that the probe bound in a calcium-dependent manner to all three proteins (supplementary fig. S6, Supplementary Material online). We could then measure fluorescence polarization of the probe peptide as a function of increasing concentrations of unlabeled peptide. A peptide that binds at the same site as the probe will displace the probe and thereby change the fluorescence polarization.

To validate the approach, we measured the binding of unlabeled probe peptide—that is, the competitor peptide we used in the phage display experiment—to hA5, hA6, and ancA5/A6 (fig. 3*A*). All three proteins are bound to the peptide. The extracted *K*_D_ values were consistent with *K*_D_ values for these protein/peptide pairs previously measured by isothermal titration calorimetry (ITC) (Wheeler et al. 2018). The new values versus the ITC values were: 2.6±2 versus 2.0±0.5 μM (hA5), 12.4±2 versus 5.0±0.5 μM (hA6), and 6.0±2 versus 1.6 ± 0.5 μM (ancA5/A6).

We next measured the binding of the nine phage display peptides to hA5, hA6, and ancA5/A6, giving 27 total peptide/protein pairs. Figure 3*A* shows representative binding curves for two different peptides to hA5. One binds with $K_{D}=46\pm 2\;\mu M$ (fig. 3*B*, black points); the other exhibits no detectable binding, implying a $K_{D}>1\;\text{mM}$ (fig. 3*B*, purple points). Of the 27 peptide/protein pairs, 17 exhibited detectable binding. We constructed a contingency table relating a peptide’s binding (yes/no) to its *E* cutoff (above/below) (fig. 3*C*). This revealed that having an E≤−1.5 strongly predicts whether binding will be detectable ($\chi_{2}:\;p=0.008;\;\phi =0.51$). Representative binding curves for hA5, hA6, and ancA5/A6 are shown for all nine peptides in supplementary figs. S7–S9, Supplementary Material online, respectively.

We wanted to know whether the enriching peptides had *K*_D_ values in a biologically relevant regime. The average *K*_D_ for peptides with E≤−1.5 was 56 μM; the best *K_D_* was 19 μM (hA5 binding to GWLEQYFSRTADGGSAE); the weakest *K*_D_ was 130 μM (ancA5/A6 binding to RHGFLQDILFKLGGSAE). These are comparable to the *K*_D_ values for the biological targets of these proteins. hA6 binds its target peptides from Annexin I and SIP with *K*_D_ values of 17 and 26 μM, respectively (Streicher et al. 2009), while hA5 binds a peptide from its putative biological target of NCX1 with $K_{D}=18\;\mu M$ (Wheeler et al. 2018).

Finally, we also investigated the quantitative relationship between $log_{10}(K_{D})$ and *E* for the 17 experiments that yielded binding curves for which we could extract *K*_D_. We observed the expected inverse relationship between affinity and *E* for the three proteins studied indvidually, as well as the combination of all three proteins (fig. 3*D*). The correlation was excellent for the five peptides binding to hA5 ($R^{2}=84.4$); however, the correlation for hA6, ancA5/A6, and the pooled samples was much worse ($R^{2}=16$). The poor correlation is due to two peptides (one binding hA6, one binding ancA5/A6) that have highly negative *E*, but low binding affinity. Taken together, this analysis suggests there is some quantitative information about binding affinity encoded in *E*, but that it is likely best viewed as a classifier: peptides with E≤−1.5 are predicted to bind with *K*_D_ values around 50 μM.

### hA5 and hA6 Did Not Increase in Specificity Relative to the Ancestor

We were now in a position to determine how specificity changed over time for these proteins. We constructed a single data set containing the 30,533 peptides for which we had measurable values of *E* for all four proteins (hA5, hA6, ancA5/A6, and altAll). If the specificity of hA5 and hA6 increased over time, we would expect them to bind to a smaller number of peptides than their ancestor. A more stringent interpretation of the increasing specificity hypothesis might further state that the set of peptides bound by hA5 and/or hA6 should largely be a subset of the peptides bound by the ancestor (fig. 1*C*).

**Fig. 4.**
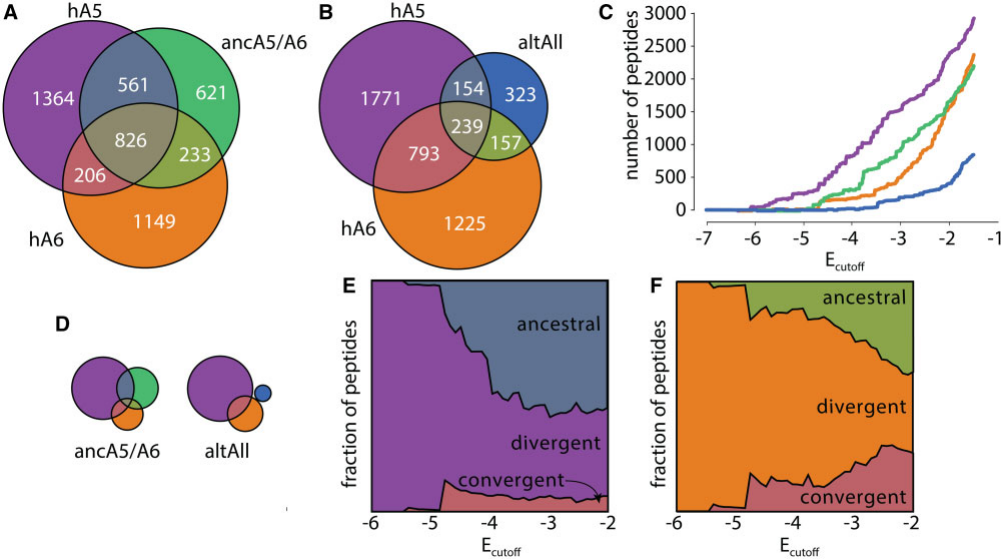
hA5 and hA6 gained new targets since their last common ancestor. (*A*) Venn diagrams for overlap between number peptides observed for hA5 (purple), hA6 (orange), and ancA5/A6 (green). The areas are proportional to the number of peptides. The absolute numbers are indicated on the plot. (*B*) Venn diagram for hA5, hA6, and altAll. Colors and markers as in (*A*). (*C*) Number of peptides observed as a function of *E*_cutoff_ for hA5 (purple), hA6 (orange), ancA5/A6 (green), and altAll (blue). (*D*) Venn diagrams for ancA5/A6 and altAll for $E_{\text{cutoff}}=-4.5$. The areas are on the same scale as (*A* and *B*). (*E*) Fraction of hA5 targets that overlap with ancA5/A6 (“ancestral”; slate), overlap with neither ancA5/A6 nor hA6 (“divergent”; purple), or overlap with hA6 but not the ancestor (“convergent”; salmon). (*E*) Fraction of hA6 targets that overlap with ancA5/A6 (“ancestral”; green), overlap with neither ancA5/A6 nor hA5 (“divergent”; orange), or overlap with hA5 but not the ancestor (“convergent”; salmon).

We tested these hypotheses by constructing a Venn diagram for all peptides with E≤−1.5 for hA5, hA6, and ancA5/A6 (fig. 4*A*). We found the hA5 set was larger than the ancA5/A6 set (2,957 vs. 2,241 peptides), while the hA6 set was essentially the same size as the ancA5/A6 set (2,414 vs. 2,241 peptides). Further, rather than hA5 and hA6 binding subsets of ancA5/A6, both bound to more unique peptides than peptides that overlapped with ancA5/A6 (fig. 4*A*). We next asked whether this was robust to phylogenetic uncertainty by calculating a Venn diagram for hA5, hA6, and altAll. The Venn diagram for the alternate ancestral reconstruction was similar to that for ancA5/A6: hA5 and hA6 did not gain specificity relative to the ancestor (fig. 4*B*). Indeed, hA5 and hA6 are less specific than altAll, as altAll binds to a much smaller number of peptides than ancA5/A6.

Our analysis in figure 4*A* and *B* showed that hA5, hA6, and ancA5/A6 bound to similar numbers of targets. We next asked if this result was robust to our choice of *E* cut-off. If we made the cutoff more stringent (more negative), would we see a change in the pattern of specificity? Figure 4*C* shows the number of peptides bound by each protein as a function of *E*_cutoff_. We found that hA5 binds to a larger set of peptides than ancA5/A6 for all values of *E*_cutoff_. In contrast, for an $E_{\text{cutoff}}<-2$, hA6 does bind to fewer peptides than ancA5/A6. Thus, with a more stringent cutoff, hA6 gained moderate specificity relative to the ancestral protein. This gain in specificity consists of hA6 binding new peptides, however, not hA6 binding a limited subset of the ancestral peptides. This can be seen in fig. 4*D*, which shows the Venn diagrams hA5, hA6, and ancA5/A6 (left) and hA5, hA6, and altAll (right) using an *E*_cutoff_ of –4.5. hA6 has acquired new peptides relative to either ancestor, even as it has a smaller overall number of peptides than ancA5/A6.

Finally, we wanted to ask whether including the “missing” responsive peptides with −1.5<E<0 would change our conclusions about the specificity of these proteins. Peptides in this range of *E* were depleted by competitor, but could not be confidently identified because they overlapped with the unresponsive distribution (fig. 2*D*). Although we could not identify the specific peptides that had *E* in this range, we did know their distribution. Using the mean and standard deviation of the responsive distribution for each protein, we could therefore calculate the fraction of responsive peptides with E≤−1.5 and the fraction with −1.5<E<0. Since we knew the number of peptides with E≤−1.5, we could thus estimate how many peptides, total, had *E *<* *0. hA5, for example, had a responsive distribution with a mean of –0.98 and a standard deviation of 2.86. With this distribution, 8% of peptides had E≤−1.5 and 7% had −1.5<E<0. There were 2,957 peptides with E≤−1.5, implying that there were 2,648 peptides with −1.5<E<0. Thus, we would estimate that hA5 had 5,604 peptides with *E *<* *0 in this experiment.

Using this approach, we estimated the number of peptides with *E *<* *0 for all four proteins. hA5 and hA6 had 5,604 and 7,186 peptides, while ancA5/A6 and altAll had 5,172 and 3,293 peptides. We thus found no evidence that hA5 and hA6 had increased specificity relative to the ancestor.

### hA5 and hA6 Exhibit Diverging Specificity

This analysis revealed that hA5, hA6, and ancA5/A6 bind to small, but overlapping, sets of all possible binding peptides (fig. 4*A*). We next set out to better understand the nature of the historical evolutionary changes. We split the interaction targets of hA5 and hA6 into three categories: ancestral (peptides shared with ancA5/A6), convergent (peptides shared between hA5 and hA6, but not the ancestor), and divergent (peptides that are not shared by any others). We then plotted this for hA5 and hA6 as a function of *E*_cutoff_ (fig. 4*E* and *F*).

We found that hA5 gave a clear pattern of divergent evolution (fig. 4*E*). If we look at moderately enriching peptides (−4.0≤E≤−1.5) we find that ≈50% of hA5’s peptides are ancestral, ≈45% are divergent, and ≈5% are convergent. For the highest enriching peptides (E<−4.0), we see the fraction of divergent peptides climbs even higher; for E≤−5.3, all hA5 peptides were unique and thus—apparently—divergent.

**Fig. 5.**
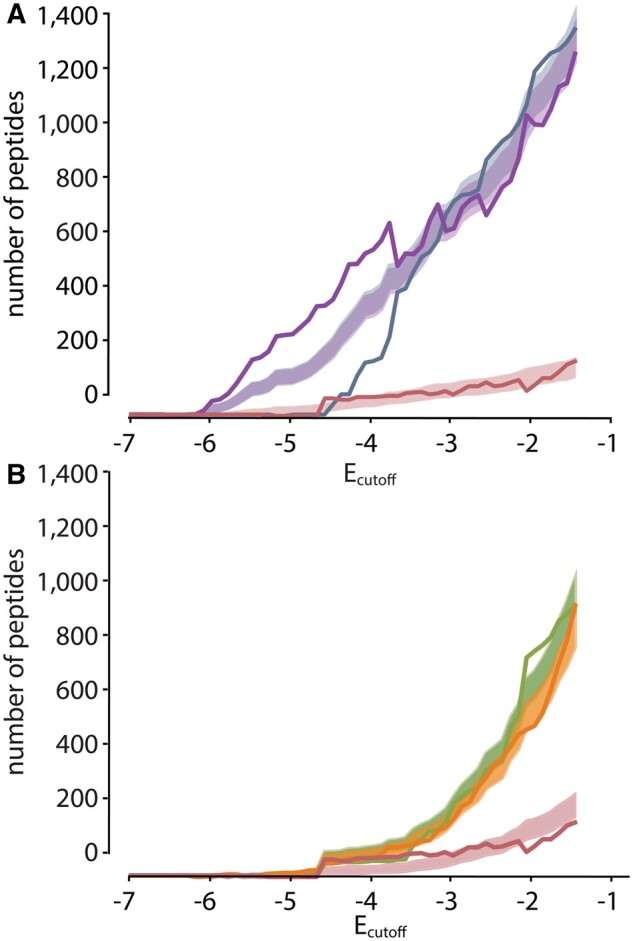
hA5 gained more highly enriched peptides since ancA5/A6. (*A*) Cumulative number of hA5 peptides observed at or below each enrichment level that are ancestral (slate), divergent (purple), or convergent (salmon). The solid lines were observed experimentally (seen in fig. 3*C*). The shaded regions indicate the 95% confidence intervals for the number of expected peptides if the underlying proportions were ancestral (0.5), divergent (0.45), and convergent (0.05). For E<−4.0, the observed number of divergent peptides is elevated above and the observed number of ancestral peptides is depressed below the expectation. (*B*) Equivalent plot for hA6. Curves are ancestral (green), divergent (orange), and convergent (salmon). There is no evidence for elevated numbers of divergent, highly enriched peptides for hA6.

One explanation for this observation is a lack of sufficient sampling: maybe the overall fraction of divergent peptides is constant across *E* values, but that the low numbers of peptides with low values of *E* led to a chance over-representation of divergent peptides. To probe for this possibility, we assumed that hA5 had populations like those reflected for moderate enrichment (ancestral: 50%, divergent: 45%, and convergent: 5%). We then walked down *E* and sampled the appropriate numbers of peptides for each *E*. (For example, we sampled a total of ≈600 hA5 peptides for an *E*_cutoff_ of –4 and only ≈100 hA5 peptides for an *E*_cutoff_ of –5.5). This allowed us to calculate the expected variation in the numbers of peptides in the ancestral, divergent, or convergent categories due to sampling. We found that the observed increase in the relative number of divergent peptides could not be explained by sampling for hA5 ($p=6.3\times 10^{-44}$ for E≤−5.5; fig. 5*A*). This indicates that hA5 has acquired an increased number of highly enriching peptides relative to its ancestral state.

We next turned our attention to hA6. Similar to hA5, it gave a pattern of divergent evolution for moderately enriching peptides (≈50% ancestral, ≈40% divergent, ≈10% convergent) and then climbed to apparently 100% divergent for the most negative values of *E* (fig. 4*F*). To see if this could be explained by the small numbers of peptides observed at these values of *E*, we repeated the sampling analysis we performed for hA5 for hA6. For hA6, we saw no evidence that the relative proportions of divergent, convergent, and ancestral categories changed as a function of enrichment (fig. 5*B*). Thus, we cannot resolve whether hA6 has acquired a relatively higher proportion of highly enriching targets since ancA5/A6.

Finally, we asked if our results were robust across biological replicates. For each biological replicate, we started from the raw reads and worked all the way through calculating the Venn diagram in figure 4*A* and the patterns of evolutionary divergence for fig. 4*E* and *F*. We obtained very similar results with both biological replicates (supplementary fig. S10, Supplementary Material online), suggesting these findings are not the result of a replicate-specific artifact.

Overall, this analysis has revealed that on both lineages, and with two different versions of the reconstructed ancestor, we see that hA5 and hA6 did not gain specificity relative to their ancestral protein. Both lineages maintained interactions with a large number of ancestral protein targets, but also gained a set of new targets. Many of the newly acquired targets were specific to hA5 or hA6, respectively, suggesting a pattern of divergent evolution. The set of partners shifted and grew (hA5) or shifted and remained the same size (hA6); neither lineage gave a pattern of simple increasing specificity over time.

## Discussion

In this work, we combined ancestral sequence reconstruction with a high-throughput assay to measure evolutionary changes in the specificity of the proteins hA5 and hA6. In a previous study of the biological binding partners of the modern proteins, we found that specificity increased since the last common ancestor of the proteins (fig. 1). In this study, we found the opposite: hA5, in particular, became slightly less specific over the same evolutionary interval (fig. 4).

These results can be rationalized if we make our definition of specificity more precise. Specificity may be viewed at two levels: *biological* and *intrinsic.* Biological specificity measures the ability of a protein to parse its biological environment. It is determined by both the affinity of a protein for its potential targets and the biological concentrations of the protein and its targets. Such specificity can be tuned by selection, as altering biological interactions can have a profound effect on an organism’s fitness.

Intrinsic specificity, in contrast, measures the affinity of the protein for all possible targets, regardless of whether a given target is encountered by the protein in a biological context. As a whole, intrinsic specificity is invisible to selection: a mutation that only alters the ability of a protein to interact with a partner it never encounters will not affect fitness. These latent, promiscuous, interactions can, however, set up future evolutionary change because the pre-existing binding interaction can be exploited for new biological functionality (Khersonsky and Tawfik 2010; Copley 2012).

A protein with low intrinsic specificity may have a higher latent capacity to form new interactions, potentially making it more “evolvable.” If proteins indeed tend to gain intrinsic specificity over time, one could even argue that they tend to become less evolvable. This would be a striking evolutionary trend (Risso et al. 2013, 2014). Practically, it would also be a strong argument for using reconstructed ancestral proteins as the starting point for protein engineering: a protein with lower intrinsic specificity would have a greater number of latent interactions to exploit and optimize (Khersonsky and Tawfik 2010; Risso et al. 2018).

Ancestral sequence reconstruction studies have, however, generally probed changes in biological rather than intrinsic specificity (Carroll et al. 2008; Eick et al. 2012; Risso et al. 2013, 2014; Pougach et al. 2014; Zou et al. 2015; Clifton and Jackson 2016; Devamani et al. 2016; Ma et al. 2016; Rauwerdink et al. 2016; Alhindi et al. 2017; Wheeler et al. 2018). Take our previous study on the evolution of S100A5 and S100A6 (Wheeler et al. 2018). We selected known partners of S100A5 and S100A6, and then asked whether those partners interacted with ancA5/A6. We found that ancA5/A6 bound all of the modern partners, indicating that S100A5 and S100A6 did, indeed, acquire new biological specificity relative to their ancestor (fig. 1).

The current study, however, reveals that increased biological specificity need not imply increased intrinsic specificity. We found that human S100A5 and S100A6 both acquired many new binding partners over the same interval they acquired new biological specificity. Human S100A5, in particular, binds to *more* targets than its ancestor: it’s intrinsic specificity decreased (fig. 3). This difference between our results for biological and intrinsic specificity suggests we must carefully define which form of specificity is under discussion when thinking about global evolutionary trends.

Do we expect a global trend in intrinsic specificity? In our view, the answer is “no.” Because intrinsic specificity is not under selection, the intrinsic specificity of the protein will be determined by chance as the protein meanders through sequence space. Presumably, many more sequences encode low intrinsic specificity than high intrinsic specificity proteins, just as many more sequences encode low stability rather than high stability proteins (Taverna and Goldstein 2002). As a result, we would expect most evolutionary steps to decrease, rather than increase intrinsic specificity.

Given this, intrinsic specificity would only increase if it was somehow linked to some other feature under selection. One might imagine, for example, that increasing biological specificity necessarily increases intrinsic specificity for certain classes of ligands. It is, however, not obvious that this will generally hold true. A mutation that changes biological specificity alters the chemistry of a protein’s binding interface, excluding some intrinsic partners, and adding others. We see no reason to assume the number of partners excluded would be systematically higher than the number added for most classes of mutations and binding sites.

Our work does not rule out a trend of increased intrinsic specificity over deep evolutionary time, but it does caution against interpreting changes in biological specificity as evidence for an overall trend. Testing for a global trend in intrinsic specificity will require studies of unbiased sets of possible interaction partners. It will also necessitate studies of multiple protein families, over deeper evolutionary time scales, and with different classes of binding partners and substrates.

## Materials and Methods

All of our raw data, as well as the scripts necessary to reproduce the work have been published in the following repository: https://github.com/harmslab/were-anc-less-specific.

### Molecular Cloning, Expression, and Purification of S100 Proteins

Proteins were expressed in a pET28/30 vector containing an N-terminal His tag with a TEV protease cleavage site (Millipore). For each protein, expression was carried out in Rosetta *E.coli* (DE3) pLysS cells. 1.5 L cultures were inoculated at a 1:100 ratio with saturated overnight culture. *E. coli* were grown to high log-phase (OD_600_ = 0.8–1.0) with 250 rpm shaking at 37°C. Cultures were induced by addition of 1 mM IPTG along with 0.2% glucose overnight at 16°C. Cultures were centrifuged and the cell pellets were frozen at 20°C and stored for up to 2 months. Lysis of the cells was carried out via sonication on ice in 25 mM Tris, 100 mM NaCl, 25 mM imidazole, pH 7.4. The initial purification step was performed at 4°C using a 5 mL HiTrap Ni-affinity column (GE Health Science) on an Äkta PrimePlus FPLC (GE Health Science). Proteins were eluted using a 25 mL gradient from 25 to 500 mM imidazole in a background buffer of 25 mM Tris, 100 mM NaCl, pH 7.4. Peak fractions were pooled and incubated overnight at 4°C with ≈1:5 TEV protease (produced in the lab). TEV protease removes the N-terminal His-tag from the protein and leaves a small Ser-Asn sequence N-terminal to the wild-type starting methionine. Next, hydrophobic interaction chromatography (HIC) was used to purify the S100s from remaining bacterial proteins and the added TEV protease. Proteins were passed over a 5 mL HiTrap phenyl-sepharose column (GE Health Science). Due to the ${\text{Ca}}^{2+}$-dependent exposure of a hydrophobic binding, the S100 proteins adhere to the column only in the presence of ${\text{Ca}}^{2+}$. Proteins were pre-saturated with 2 mM ${\text{Ca}}^{2+}$ before loading on the column and eluted with a 30 mL gradient from 0 mM to 5 mM EDTA in 25 mM Tris, 100 mM NaCl, pH 7.4.

Peak fractions were pooled and dialyzed against 4 L of 25 mM Tris, 100 mM NaCl, pH 7.4 buffer overnight at 4°C to remove excess EDTA. The proteins were then passed once more over the 5 mL HiTrap Ni-affinity column (GE Health Science) to remove any uncleaved His-tagged protein. The cleaved protein was collected in the flow-through. Finally, protein purity was examined by SDS-PAGE. If any trace contaminants appeared to be present, we performed anion chromatography with a 5 mL HiTrap DEAE column (GE). Proteins were eluted with a 50 mL gradient from 0-500 mM NaCl in 25 mM Tris, pH 7.4 buffer. Pure proteins were dialyzed overnight against 2 L of 25 mM TES (or Tris), 100 mM NaCl, pH 7.4, containing 2 g Chelex-100 resin (BioRad) to remove divalent metals. After the final purification step, the purity of protein products was assessed by SDS PAGE and MALDI-TOF mass spectrometry to be >95%. Final protein products were flash frozen, dropwise, in liquid nitrogen and stored at −80°C. Protein yields were typically on the order of 25 mg/1.5 L of culture.

### Preparation of Biotinylated Proteins for Phage Display

A mutant version of hA5 with a single N-terminal Cys residues was generated via site-directed mutagenesis using the QuikChange lightning system (Agilent). The Cys was introduced in the Ser–Asn tag leftover from TEV protease cleavage as Ser–Asn–Cys. The proteins were expressed and purified as described in the previous section. A small amount of the purified proteins were biotinylated using the EZ-link BMCC-biotin system (ThermoFisher Scientific). ≈1 mg BMCC-biotin was dissolved directly in 100% DMSO to a concentration of 8 mM for labeling. Proteins were exchanged into 25 mM phosphate, 100 mM NaCl, pH 7.4 using a Nap-25 desalting column (GE Health Science) and degassed for 30 min at 25°C using a vacuum pump (Malvern Instruments). While stirring at room temperature, 8 mM BMCC-biotin was added dropwise to a final 10× molar excess. Reaction tubes were sealed with PARAFILM (Bemis), and the maleimide-thiol reactions were allowed to proceed for 1 h at room temperature with stirring. The reactions were then transferred to 4°C and incubated with stirring overnight to allow completion of the reaction. Excess BMCC-biotin was removed from the labeled proteins by exchanging again over a Nap-25 column (GE Health Science), and subsequently a series of three concentration-wash steps on a NanoSep 3 K spin column (Pall corporation), into the Ca-TeBST-loading buffer. Complete labeling was confirmed by MALDI-TOF mass spectrometry by observing the ≈540Da shift in the protein peak. Final stocks of labeled proteins were prepared at 10 µM by dilution into the loading buffer.

### Phage Display

Phage display experiments were performed using the PhD-12 peptide phage display kit (NEB). All steps involving the pipetting of phage-containing samples was done using filter tips (Rainin). We prepared 100 μL samples containing phage ($5.5\times 10^{11}$ PFU) and 0.01 μM biotin-protein (or biotin alone in the negative control) at room temperature in a background of ${\text{Ca}}^{2+}$-TeBST loading buffer (50 mM TES, 100 mM NaCl, 2 mM CaCl_2_, 0.01% Tween-20, pH 7.4) to ensure ${\text{Ca}}^{2+}$-saturation of the S100 proteins. For the experiments using a peptide competitor, we included the peptide RSHSGFDWRWAMEALTGGSAE at 20 μM in the loading buffer. This peptide (named A6cons in the original report), binds all four proteins at the canonical binding site with *K*_D_ between 1 and 8  μM (Wheeler et al. 2018). Samples were incubated at room temperature for 2 h. Each sample was then applied to one well of a 96-well high-capacity streptavidin plate (previously blocked using PhD-12 kit blocking buffer and washed 6× with 150 μL loading buffer). Samples were incubated on the plate with gentle shaking for 20 min. 1 μL of 10 mM biotin (NEB) was then added to each sample on the plate and incubated for an additional five minutes to compete away purely biotin-dependent interactions. Samples were then pulled from the plate carefully by pipetting and discarded. Each well was washed 5× with 200 L of loading buffer by applying the solution to the well and then immediately pulling off by pipetting. Finally, 100 L of EDTA-TeBST elution buffer (50 mM TES, 100 mM NaCl, 5 mM EDTA, 0.01% Tween-20, pH 7.4) was applied to each well and the plate was incubated with gentle shaking for 1 h at room temperature to elute. Eluates were pulled from the plate carefully by pipetting and stored at 4 °C. Eluates were tittered to quantify eluted phage as follows. Serial dilutions of the eluates from $1:10\;\text{to}\;1:10^{5}$ were prepared in LB medium. These were used to inoculate 200 L aliquots of mid-log-phase ER2738 *E. coli* (NEB) by adding 10 L to each. Each 200 L aliquot was then mixed with 3 mL of premelted top agar, applied to a LB agar XGAL/IPTG (Rx Biosciences) plate, and allowed to cool. The plates were incubated overnight at 37 °C to allow the formation of plaques. The next morning, blue plaques were counted and used to calculate PFU/mL phage concentration. Enrichment was calculated as a ratio of experimental samples to the biotin-only negative control.

To generate the input phage library, the commercially-produced library was first screened in duplicate against each of the four proteins as described above. Each of these lineages was subsequently amplified in ER2738 *E. coli* (NEB) as follows. 20 mL 1:100 dilutions of an ER2738 overnight culture were prepared. Each 20 mL culture was inoculated with one entire sample of remaining phage eluate. The cultures were incubated at 37 °C with shaking for 4.5 h to allow phage growth. Bacteria were then removed by centrifugation, and the top 80% of the culture was removed carefully with a filtered serological pipette and transferred to a fresh tube containing 1/6 volume of PEG/NaCl (20% w/v PEG-8000, 2.5 M NaCl). Samples were incubated overnight at 4 °C to precipitate phage. Precipitated phage were isolated by centrifugation and subsequently purified by an additional PEG/NaCl precipitation on ice for 1 h. These individually amplified pools were then resuspended in 200 μL each of sterile loading buffer and mixed together to form a pre-conditioned library in order to minimize the impact of sampling on the subsequent panning experiment. The pool was diluted 1:1 with 100% glycerol and stored at −20 °C for use in the final panning experiments.

### Preparation of Deep Sequencing Libraries

Phage genomic ssDNA was isolated from leftover amplified eluates from each round of panning using the M13 spin kit (Qiagen). Products were stored in low TE buffer. These ssDNA were used as the template for two replicate PCRs with the Cs1 forward (5ʹ—ACACTGACGACATGGTT CTACAGTGGTACC TTTCTATTCTCACTCT—3ʹ) and PhD96seq-Cs2 reverse (5ʹ—TACGGTAGCAGAGACTTG GTCTCCCTCA TAGTTAGCGTAACG—3ʹ) primers. Products were isolated from these PCR reactions using the GeneJet gel extraction kit (Thermo Scientific) and pooled. The pooled products were then used as templates for a secondary reaction with the barcoded primers. Products were isolated from these final PCRs using the GeneJet gel extraction kit. Concentration of barcoded samples was measured by $A_{260}/A_{280}$ using a 1 mm cuvette on an Eppendorf biospectrometer. Multiplexing was done by mixing samples according to mass. The concentration of the multiplexed library was corrected using qPCR with the P5 and P7 Illumina flow-cell primers. The library was then diluted to a final concentration of 10 nM and Illumina sequenced on two lanes of a HiSeq 4000 instrument, using the Cs1 F’ as the R1 sequencing primer. The lanes were spiked with 20% PhiX control DNA due to the relatively low diversity of the library.

Our fastq files are available for download from the NCBI short read archive with accession PRJNA646756.

### Phage Display Analysis Pipeline

We performed quality control on three read features. First, we verified that the sequence had exactly the anticipated length from the start of the phage sequence through the stop codon. Second, we only took sequences in which the invariant phage sequence differed by at most one base from the anticipated sequence. This allows for a single point mutation and or sequencing errors, but not wholesale changes in the sequence. Finally, we took only reads with an average phred score better than 15. The vast majority of the reads that failed our quality control did not have the variable region, representing reversion to phage with a wildtype-like coat protein. This analysis is encoded in the *hops_count.py* script (https://github.com/harmslab/hops_enrich), which takes a gzipped fastq file as input and returns the counts for every peptide in the file.

### Identifying the Read Count Cutoff

One critical question is at what point the number of reads correlates with the frequency of a peptide. If we set the cutoff too low, we incorporate noise into downstream analyses. If we set the cutoff too high, we remove valuable observations from our data set. To identify an appropriate cutoff, we studied the mapping between *c_i_* (the number of reads arising from peptide *i*) and *f_i_* (the actual frequency of peptide *i* in the experiments). Our goal was to find $P(f_{i}|c_{i},N)$: the probability peptide *i* is at *f_i_* given we observe it *c_i_* times in *N* counts. Using Bayes theorem, we can write:

(2) $$P(f_{i}|c_{i},N)=\frac{P(c_{i}|f_{i},N)P(f_{i})}{P(c_{i})},$$

where *N* is the total number of reads. We calculated $P(c_{i}|f_{i},N)$ assuming a binomial sampling process: what is the probability of observing exactly *c* counts given *N* independent samples when a population with a peptide frequency *f_i_*? This gives the curve seen in supplementary fig. S2*A*, Supplementary Material online. We then estimated $\hat{P(f_{i})}$ from the distribution of frequencies in the input library, constructing a histogram of apparent peptide frequencies (supplementary fig. S2*B*, Supplementary Material online). Empirically, we found that frequencies followed an exponential distribution over the measurable range of frequencies. Finally, we assumed that all counts have equal prior probabilities, turning $P(c_{i})$ into a scalar that normalizes the integral of $P(f_{i}|c_{i},N)$ so it sums to 1.

Using the information from supplementary fig. S2*A* and *B*, Supplementary Material online, we could then calculate $\text{max}\left(P(f_{i}|c_{i},N)\right)$ for any number of reads in an experiment *N*. This corresponds to the maximum likelihood estimate of *f_i_* given we observe *c_i_* counts in *N* reads. Supplementary fig. S2C, Supplementary Material online, shows this calculation for $N=2.0\times 10^{7}$ reads—a typical number of reads from our experimental replicates. We found the curve was linear above three reads. Below this, counts no longer correlates linearly with frequency, as it is possible to obtain one or two reads from random sampling from low-frequency library members. To be conservative, we used a cutoff of six counts for all downstream analyses. In total, 74.0% of reads passed our quality control and read cutoff (supplementary table S1, Supplementary Material online).

### Measuring Enrichment Values

We next set out to measure changes in the frequency of peptides between the competitor and non-competitor samples. The simplest way to do this would be to identify peptides seen in both experiments, and then measure how their frequencies change between conditions. Unfortunately, these proteins all bind a wide swath of peptide targets, and relatively few peptides were shared between conditions. This approach would thus exclude the majority of sequences. Worse, because we are interested in peptides that are lost when competitor peptide is added, ignoring peptides with no counts in the competitor sample means ignoring some of the most informative peptides.

To solve this problem, we clustered similar peptides and measured enrichment for peptide clusters rather than individual peptides. We extracted all peptides that were observed across the competitor and noncompetitor samples for a given protein. We then used DBSCAN to cluster those peptides according to sequence similarity, as measured by their Hamming distance (Ester et al. 1996). This revealed extensive structure in our data. For example, hA5 yielded 8,645 clusters with more than one peptide, incorporating more than half of the unique peptides (supplementary fig. S3*A*, Supplementary Material online). We chose clustering parameters that led to highly similar peptides within each cluster, as can be seen by the representative sequence logos for three clusters of hA5 (supplementary fig. S3*B*, Supplementary Material online). Sequences that were not placed in clusters were treated as clusters with a size of one.

We then used the enrichment of each cluster to estimate the enrichment of individual peptides. We defined enrichment as:

(3) $$E_{\text{cluster}}=-\text{ln}\left(\frac{\sum_{i=1}^{i\leq N}\beta_{i}}{\sum_{i=1}^{i\leq N}\alpha_{i}}\right),$$

where *N* is the total number of peptides in the cluster, *β_i_* is the frequency of peptide *i* in the competitor sample, and *α_i_* is the frequency of peptide *i* in the noncompetitor sample. We then made the approximation that all members of the cluster have the same enrichment:

(4) $$E_{i}\approx E_{\text{cluster}},$$

allowing us to estimate the enrichment of all *i* peptides in the cluster (supplementary fig. S3*C*, Supplementary Material online). Peptides lost because of competition for the interface will add zeros to the numerator of equation (3), leading to an overall decrease in enrichment. Peptides missed because of finite sampling will add zeros evenly to the competitor and non-competitor samples, leading to no net enrichment.

We tested this cluster-based approximation using the 8,672 peptides of hA5 for which we could directly calculate enrichment (i.e., those peptides seen in both the competitor and noncompetitor experiments). We calculated the enrichment of each peptide individually and compared these values to those obtained by the cluster method. There is no systematic difference in the values estimated using the two methods, and the linear model explains 98.4% of the variation between the two methods.

We clustered peptides using our own implementation of the DBSCAN algorithm (Ester et al. 1996) using the Hamming distance. The main parameter for DBSCAN clustering is *ε*—the neighborhood cut-off. Clusters are defined as sequences that can be reached through a series of *ε*-step moves. We found that ε=1 gave the best results for our downstream analysis. Our whole enrichment pipeline—including clustering—can be run given a peptide count file for the noncompetitor experiment and a peptide-count file for the competitor experiment using the *hops_enrich.py* script (https://github.com/harmslab/hops_enrich).

### Peptide Binding by Fluorescence Polarization

We did the fluorescence polarization experiments in 50 mM TES, 100 mM NaCl, and 0.01% Tween-20, pH 7.4, thus matching the phage display experimental buffer. We ordered all nine test peptides and the probe peptide in milligram quantities at 95% purity (Genscript). For the probe peptide, we used “5FAM-GFDWRWGMEALTGGGSAE,” where 5FAM indicates an N-terminally linked 5-carboxyfluorescein moiety. We dissolved all peptides to 10 or 20 mM (depending on solubility) in DMSO. The only exception was SRQTTSTHEWVVGGGSAE, which we dissolved in $ddH_{2}O$.

We measured fluorescence anisotropy using a SpectraMax i3 plate reader (Molecular Devices) with 96-well black plates (Corning). Prior to reading each plate, we shook for 5 s and then acquired with 500 ms integration time. Prior to the set of experiments, we optimized detector height and calibrated the plate’s xy position to maximize signal. We then kept this calibration throughout the set of experiments. For each experiment, we recorded both vertical (*v*_v_) and horizontal (*v*_h_) emission channels and then converted to arbitrary polarization units by:

(5) $$R=\frac{v_{v}-Gv_{h}}{v_{v}+2Gv_{h}}$$

with *G* set to 1.0. Titrations were done by serial dilution through conditions on individual plates, followed by measurement of all wells. We did all measurements in technical triplicate. We used custom software to programmatically remove technical replicate outliers.

We measured probe peptide affinity for each protein by increasing protein concentration from 0 to ≈200 μM protein in the presence of $2\;\text{mM}\;{\text{CaCl}}_{2}$ and 0.03 μM probe peptide. We fit the curve to a single-site binding model:

(6) $$P([\text{protein}])∼\Delta P_{\text{max}}\frac{\left[\text{protein}\right]}{[\text{protein}]+K_{D,\text{probe}}}+P_{0}$$

where *P*_0_ is the polarization of the peptide in the absence of protein, $\Delta P_{\text{max}}$ is the change in signal from unbound peptide to bound to the protein, and $K_{D,\text{probe}}$ is the probe dissociation constant. After recording the titration curve in the presence of CaCl_2_, we added 5 mM EDTA to all wells and remeasured the curve.

For our production experiments, we added increasing amounts of competitor peptide to wells with between 2.4 and 4.2 µM* * protein (monomer). Each well also contained probe peptide at the same concentration as the protein. We selected these concentrations to maximize the amount of signal change upon addition of competitor peptide $K_{D,\text{probe}}$. We calculated the expected signal change by:

(7) $$\Delta P_{\text{predicted}}=\Delta P_{\text{max}}\times \frac{\left[\text{MX}\right]}{{\left[X\right]}_{T}}$$

where $\Delta P_{\text{max}}=0.135$ (as determined by our initial fits to extract probe peptide $K_{D,\text{probe}}$), ${\left[X\right]}_{T}$ was the total probe concentration, and [MX] was the concentration of the probe/protein complex prior to adding competitor peptide. We calculated [MX] by:

(8) $$[\text{MX}]=\frac{({\left[X\right]}_{T}{+[M]}_{T}+K_{D,\text{probe}})\pm \sqrt{{\left({\left[X\right]}_{T}{+[M]}_{T}+K_{D,\text{probe}}\right)}^{2}-4{\left[X\right]}_{T}{\left[M\right]}_{T}}}{2}$$

where ${\left[M\right]}_{T}$ was the total protein concentration.

Prior to fitting a binding model to each curve, we converted it from polarization to fractional saturation. We did so by first fitting the single-site binding model to the data to obtain an estimate of the initial polarization (*P*_0_) for the protein/peptide pair. We then subtracted this value of *P*_0_ to obtain a baseline-subtracted polarization. We then divided the baseline-subtracted polarization by $\Delta P_{\text{predicted}}$, yielding a fractional saturation (*θ*). We did not fit data for which max(θ)<0.5.

We then fit a reduced form of the single-site binding model to the data:

(9) $$\theta =\frac{\left[\text{protein}\right]}{[\text{protein}]+K_{D,\text{apparent}}}$$

Finally, we corrected the resulting $K_{D,\text{apparent}}$ to account for the effect of the competitor peptide (Hulme and Trevethick 2010):

(10) $$K_{D,\text{peptide}}=\frac{K_{D,\text{apparent}}}{1+{\left[X\right]}_{T}*(1-\delta /2)/K_{D,\text{probe}}+\delta /(1-\delta )}$$

where ${\left[X\right]}_{T}$ is the total probe peptide concentration and $\delta =[{\text{MX}]/[X]}_{T}$.

## Supplementary Material

Supplementary data are available at *Molecular Biology and Evolution* online.

## Supplementary Material

msab019_Supplementary_Data
