# Supplementary material for: Were Ancestral Proteins Less Specific?
Source: Mol Biol Evol. 2021 Feb 2;38(6):2227–39. doi: 10.1093/molbev/msab019 (PMC8136485; doi:10.1093/molbev/msab019)
Supplement: msab019_Supplementary_Data [file msab019_supplementary_data.zip › supplement.pdf]

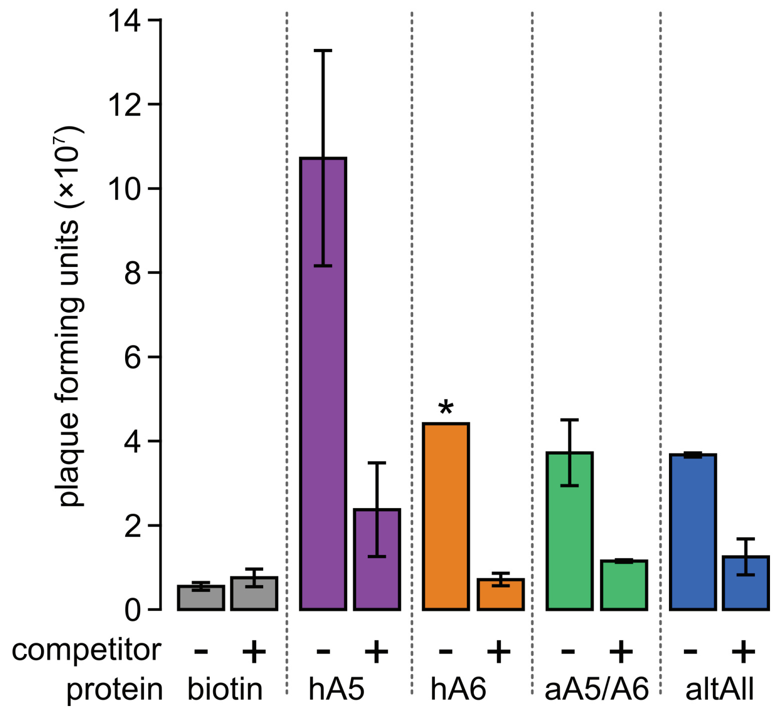

Fig S1. Phage enrichment is reduced in the presence of competitor peptide. Figure shows eluted plaque forming units (PFU) (estimated from phage titer) for two biological replicates of each condition. Enrichment is shown for biotin-only control (gray), hA5 (purple), hA6 (orange), ancA5/A6 (dark green), and altAll (light green) with (+) and without (-) 20\ \mu M competitor peptide. Error bars show the standard error for two biological replicates. (*) hA6 without competitor is shown for only one replicate due to failure of the titer for the one replicate.

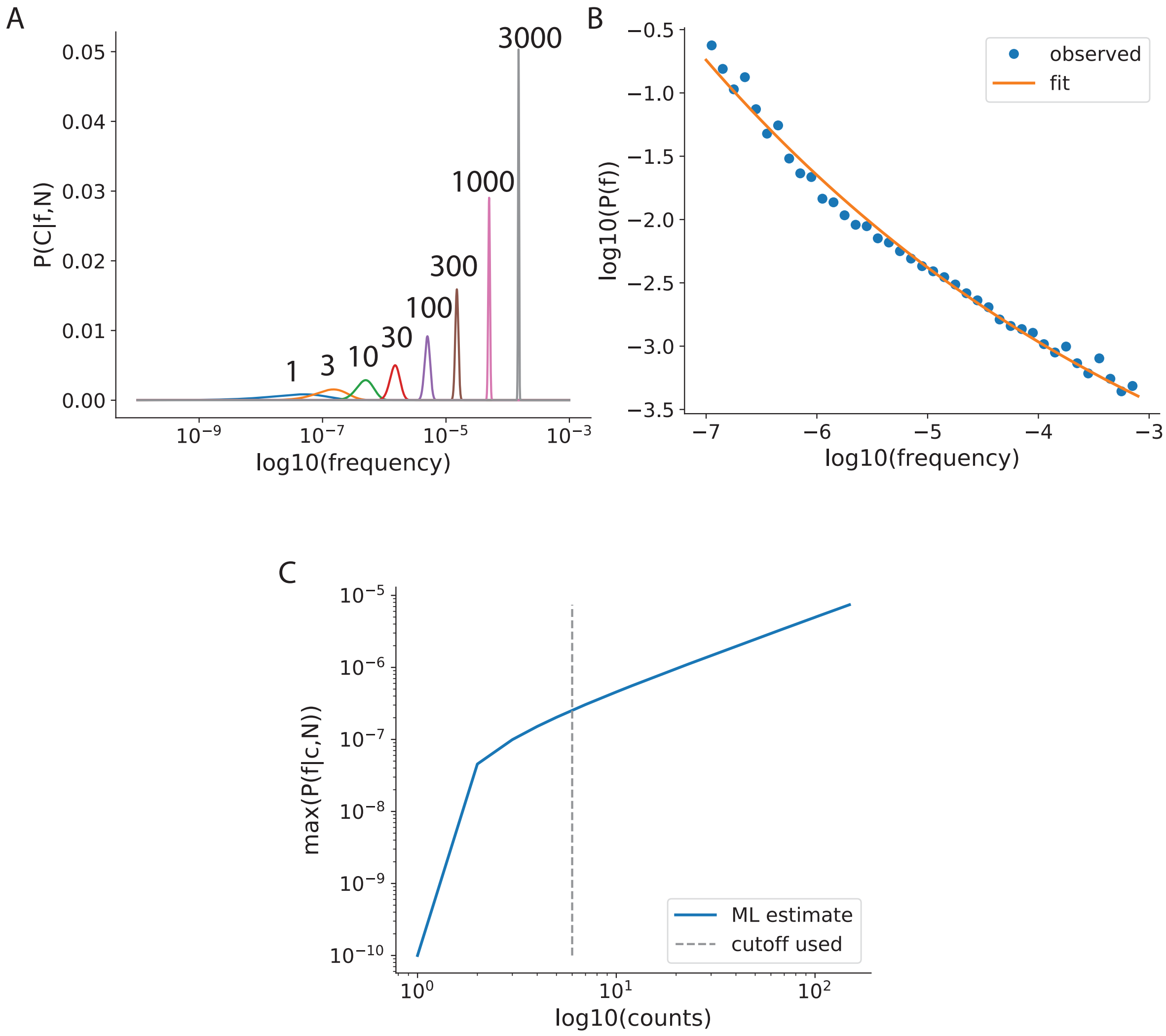

Fig S2. We can identify the number of counts that reliably reports on frequency in a sequenced phage pool. A) Using binomial sampling, we can calculate the probability of observing exactly c counts in N samples from a pool that has a peptide of actual frequency f. Figure shows curves for counts ranging from 1 To 3,000, all using N=20,000,000. B) Panel shows a histogram of frequencies estimated from 39,000,000 reads taken from the input library. The points are experimental data. The curve is an exponential distribution fit to that curve. C) Using the sampling from panel A and the fit curve from panel B, we can determine the maximum likelihood estimate of P(f|c,N). The curve shows the relationship between the number of reads for peptide i (x-axis) against the maximum-likelihood estimate of the frequency (y-axis). The gray dashed line highlights the cutoff we used in our experiments.

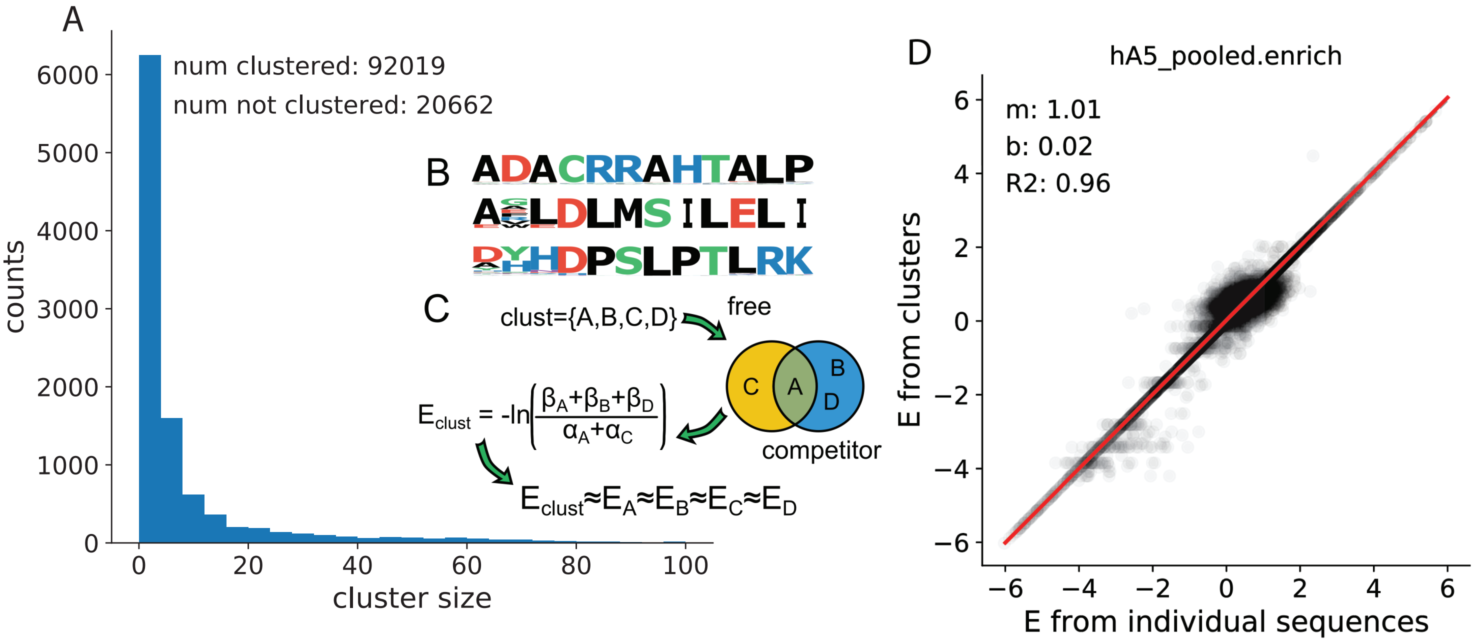

Fig S3. We can estimate how addition of competitor peptide alters the frequencies of peptides. A) Distribution of sizes of peptide clusters from hA5 experiment. Numbers indicatee how many peptides were placed into crlusters or not. B) Three example clusters taken from the clusters in panel A. The letter height at each position indicates its frequency in the sequences within that cluster. C) Toy example showing how enrichment is calculated for a cluster containing peptides A,B,C,D. Peptides A and C were observed in the no competitor sample at frequencies alpha_{A} and alpha_{C}. Peptides A, B, and D were observed in the competitor sample at frequencies beta_{A}, beta_{B} and beta_{D}. The enrichment of the cluster is given by the equation shown. All members of the cluster are then assigned E_clust. D) Comparison of enrichment values for hA5 peptides determined using a direct comparison of frequencies with and without competitor (x-axis) versus the clustering method (y-axis). Each point is an individual peptide. Red line is a least-squares regression line fit to the data. The dashed line is the 1:1 line.

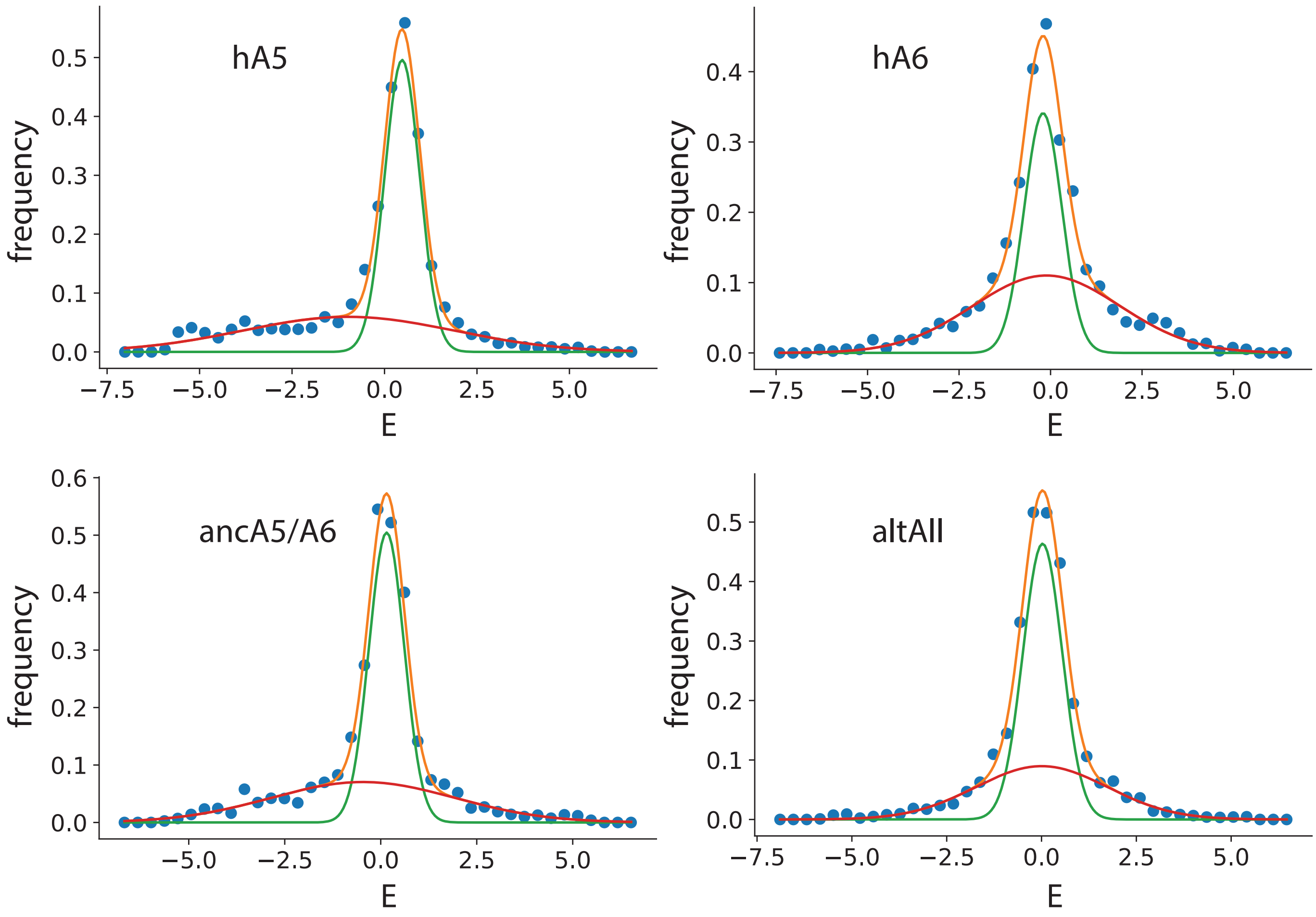

Fig S4: Histograms of enrichment values for phage display peptides. Points are bin frequencies determined from the pooled bioreplicates for each of the four proteins (hA5, hA6, ancA5/A6, and altAll). The green curve is the non-responsive distribution; the red curve is the responsive distribution; the orange curve is the sum of these two distributions.

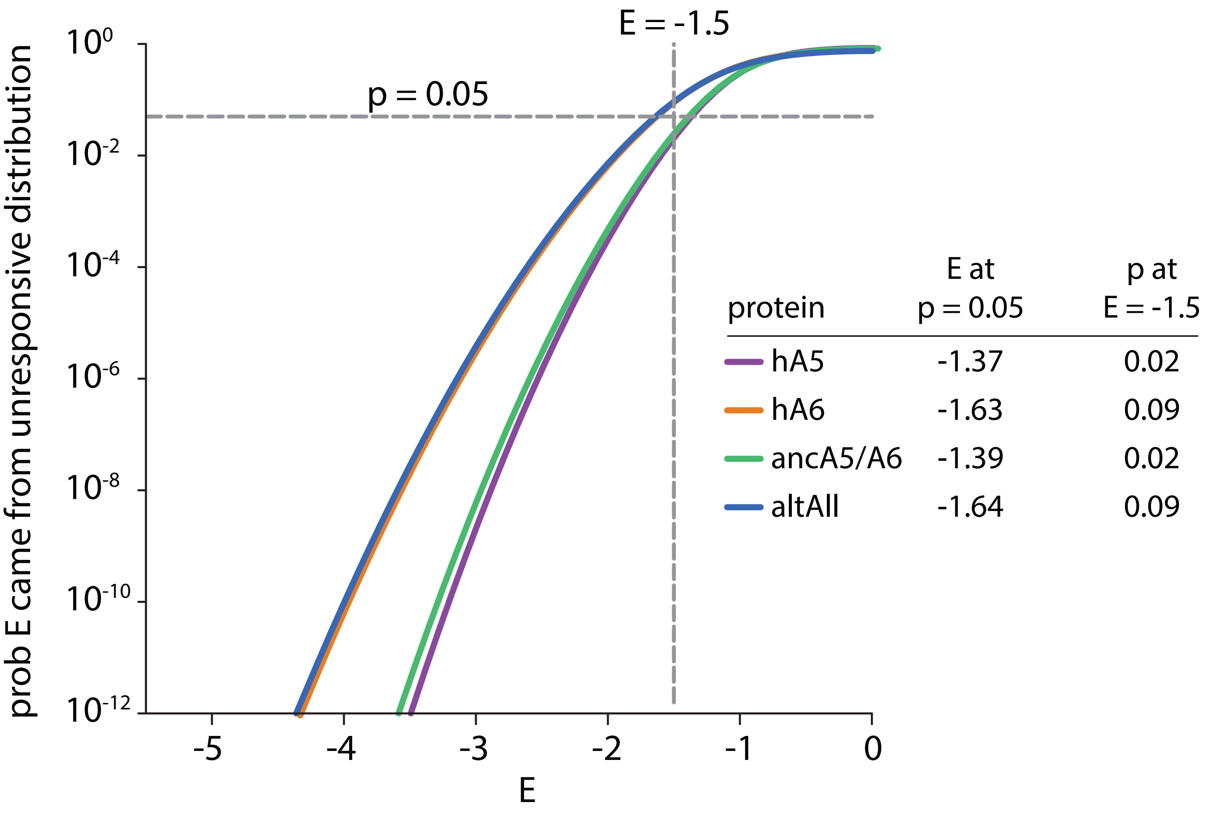

Fig S5. Selection of E=-1.5 as a cutoff for peptide enrichment. Curves show the posterior probability a peptide with the enrichment score shown on the x-axis arose from the "unresponsive" distribution for each protein. These curves were calculated by P_{unresponsive}(E)=pdf_{unresponsive}(E)/(pdf_{responsive}(E)+pdf_{unresponsive}(E)) where pdf is the probability density function in E for the normal distributions seen in Fig 2C and S4. The inset table shows the E value where the P_{unresponsive}(E) is 0.05 and the P_{unresponsive}(E) at E=-1.5.

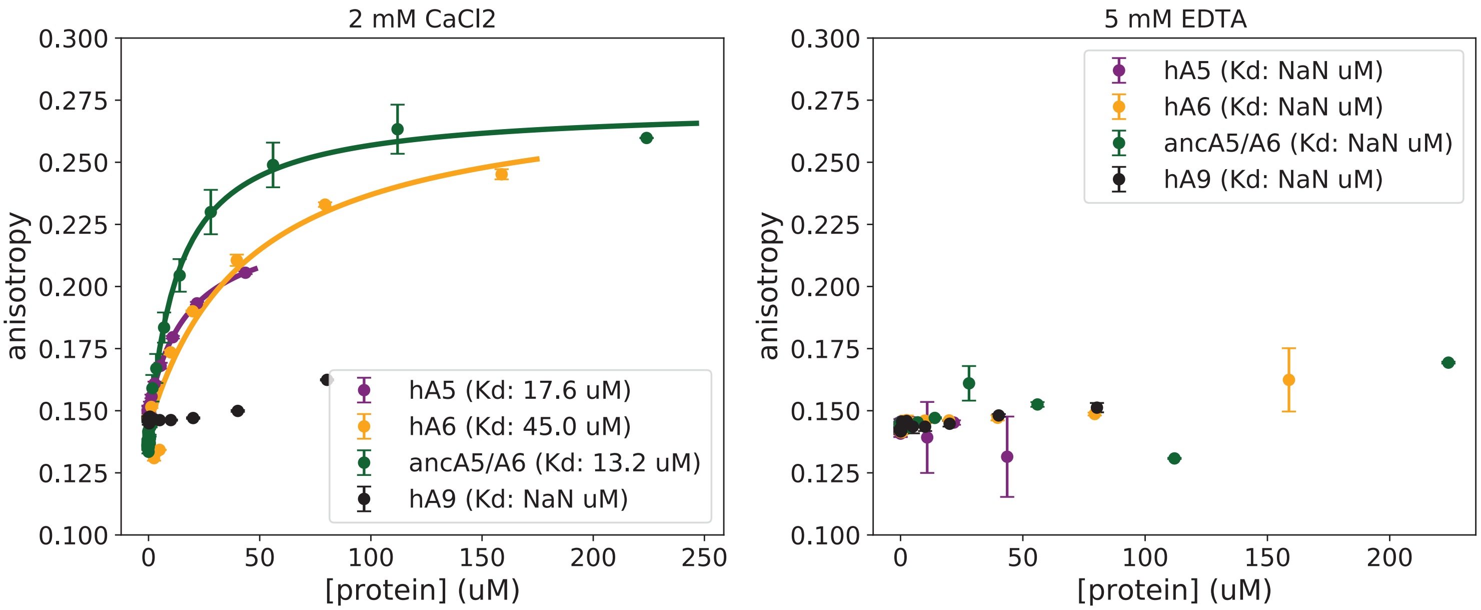

Fig S6: Binding of fluorescent probe peptide is calcium dependent. Panel show measured fluorescence anisotropy for the peptide 5FAM-GFDWRWGMEALTGGGSAE in the presence of increasing protein concentration. Probe concentration was 0.03 uM. Proteins are hA5 (purple), hA6 (orange), ancA5/A6 (green), and human S100A9 (black). S100A9 is a negative control, because it is evolutionarily distant from hA5 and hA6 and would thus not be expected to bind to the probe peptide. Panel on the left shows binding in the presence of 2 mM CaCl2. Curves are fit to a single-site binding model, with apparent KD for each protein in the legend. Right panel show the same samples with 5 mM EDTA added. Binding is eliminated for all samples.

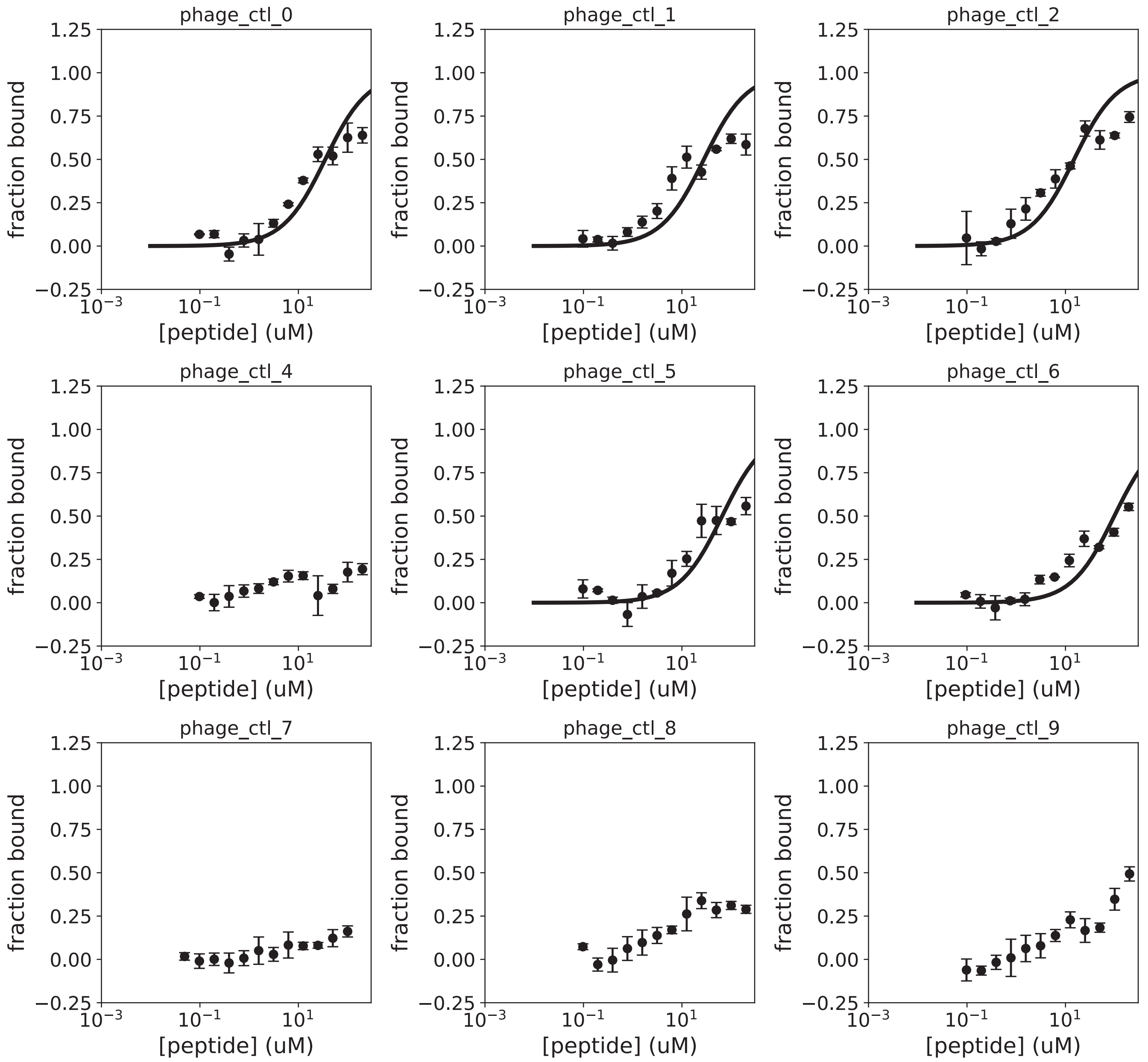

Fig S7: binding of phage peptides to hA5 by fluorescence polarization.

phage_ctl_0 EGLDLMSILELIGGGSAE
phage_ctl_1 RHGFLQDILFKLGGGSAE
phage_ctl_2 GWLEQYFSRTADGGGSAE
phage_ctl_4 SRQTTSTHEWVVGGGSAE
phage_ctl_5 EQPLLKYLQLMRGGGSAE
phage_ctl_6 HVQWRDRNVIEWGGGSAE
phage_ctl_7 GEVTNYGYLVDQGGGSAE
phage_ctl_8 SSSTYPGFRQSTGGGSAE
phage_ctl_9 SGPSDWLHKGVLGGGSAE

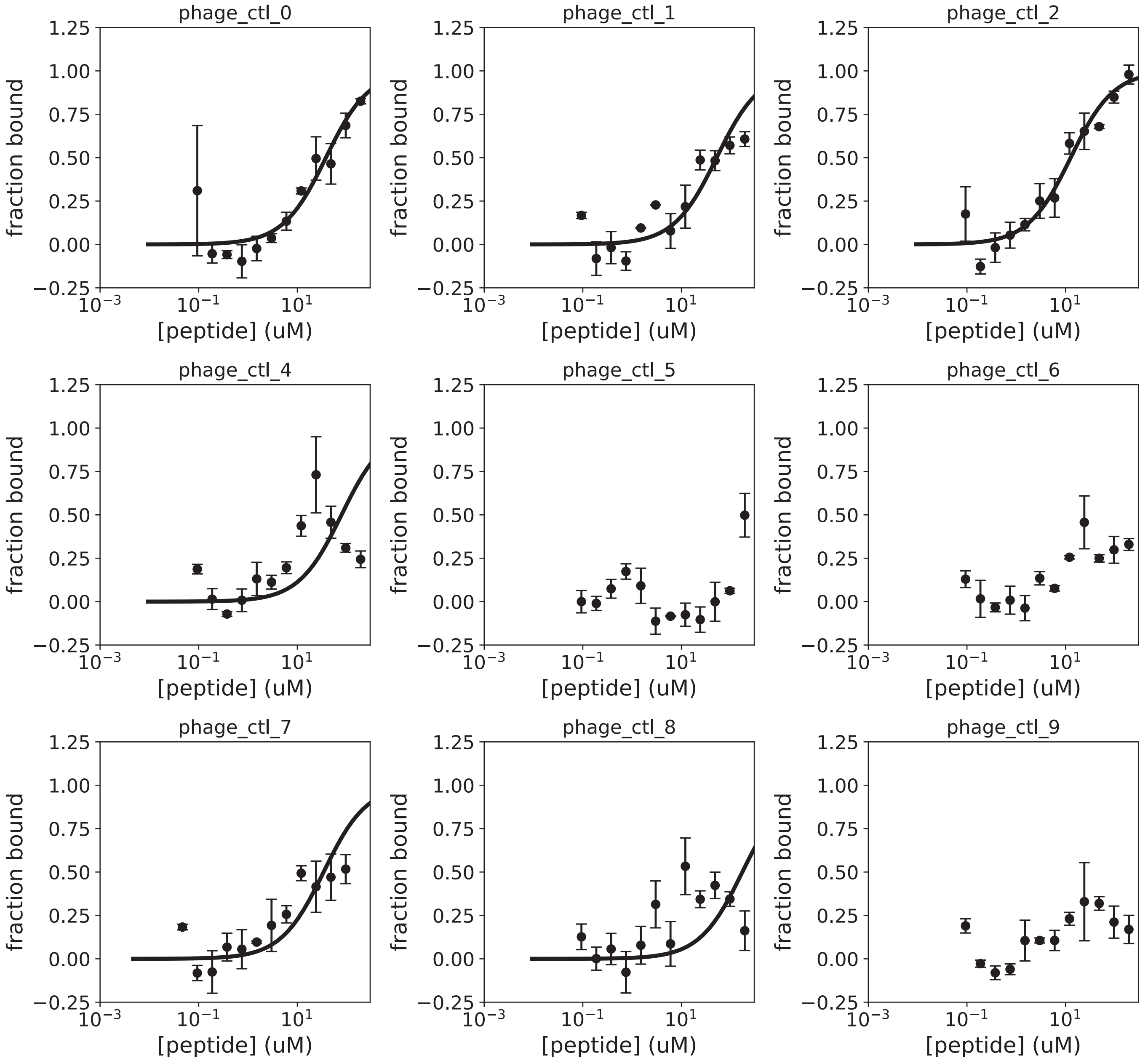

Fig S8: binding of phage peptides to hA6 by fluorescence polarization.

phage_ctl_0 EGLDLMSILELIGGGSAE
phage_ctl_1 RHGFLQDILFKLGGGSAE
phage_ctl_2 GWLEQYFSRTADGGGSAE
phage_ctl_4 SRQTTSTHEWVVGGGSAE
phage_ctl_5 EQPLLKYLQLMRGGGSAE
phage_ctl_6 HVQWRDRNVIEWGGGSAE
phage_ctl_7 GEVTNYGYLVDQGGGSAE
phage_ctl_8 SSSTYPGFRQSTGGGSAE
phage_ctl_9 SGPSDWLHKGVLGGGSAE

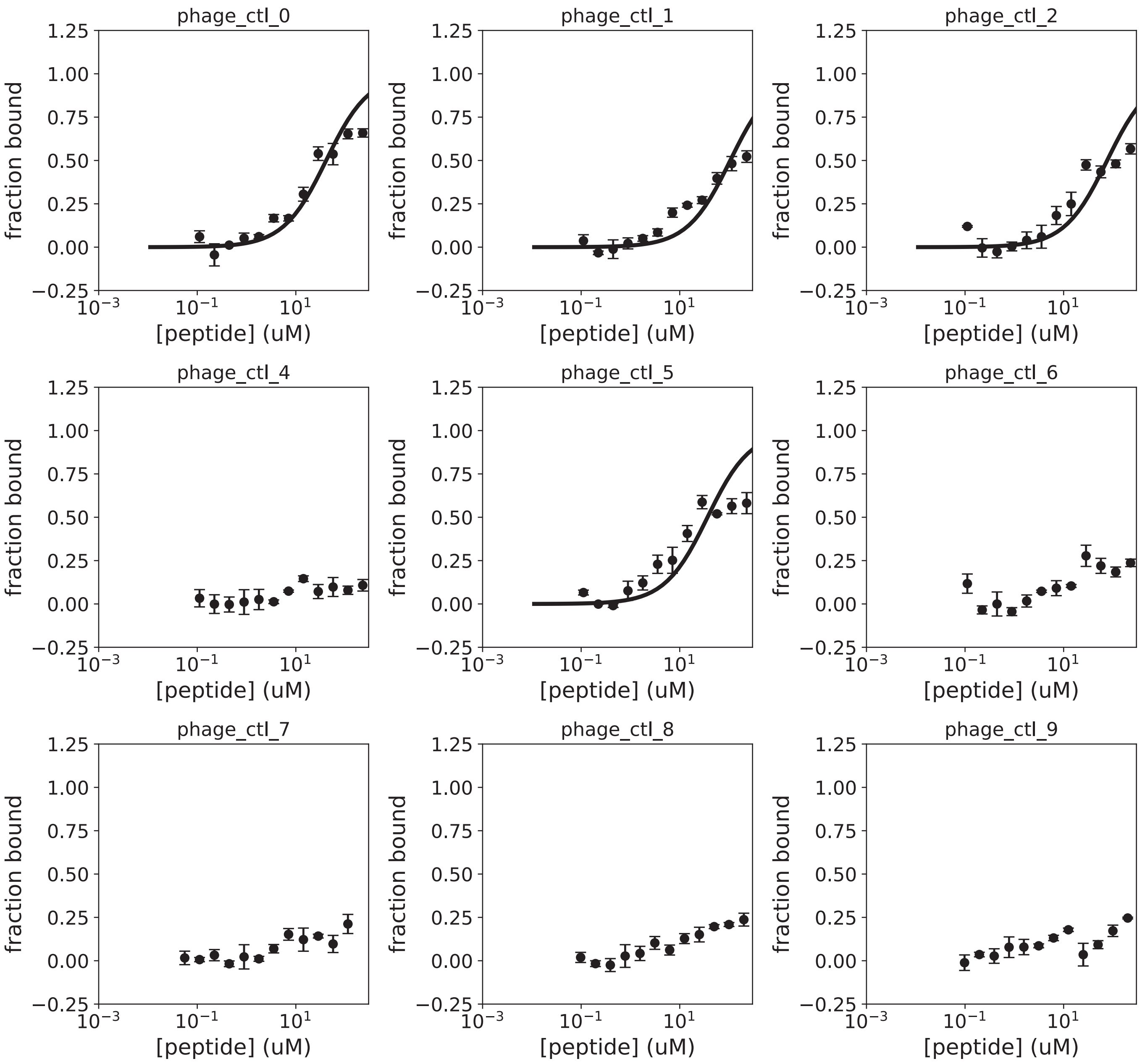

Fig S9: binding of phage peptides to hA5 by fluorescence polarization.

phage_ctl_0	EGLDLMSILELIGGGSAE
phage_ctl_1	RHGFLQDILFKLGGGSAE
phage_ctl_2	GWLEQYFSRTADGGGSAE
phage_ctl_4	SRQTTSTHEWVVGGGSAE
phage_ctl_5	EQPLLKYLQLMRGGGSAE
phage_ctl_6	HVQWRDRNVIEWGGGSAE
phage_ctl_7	GEVTNYGYLVDQGGGSAE
phage_ctl_8	SSSTYPGFRQSTGGGSAE
phage_ctl_9	SGPSDWLHKGVLGGGSAE

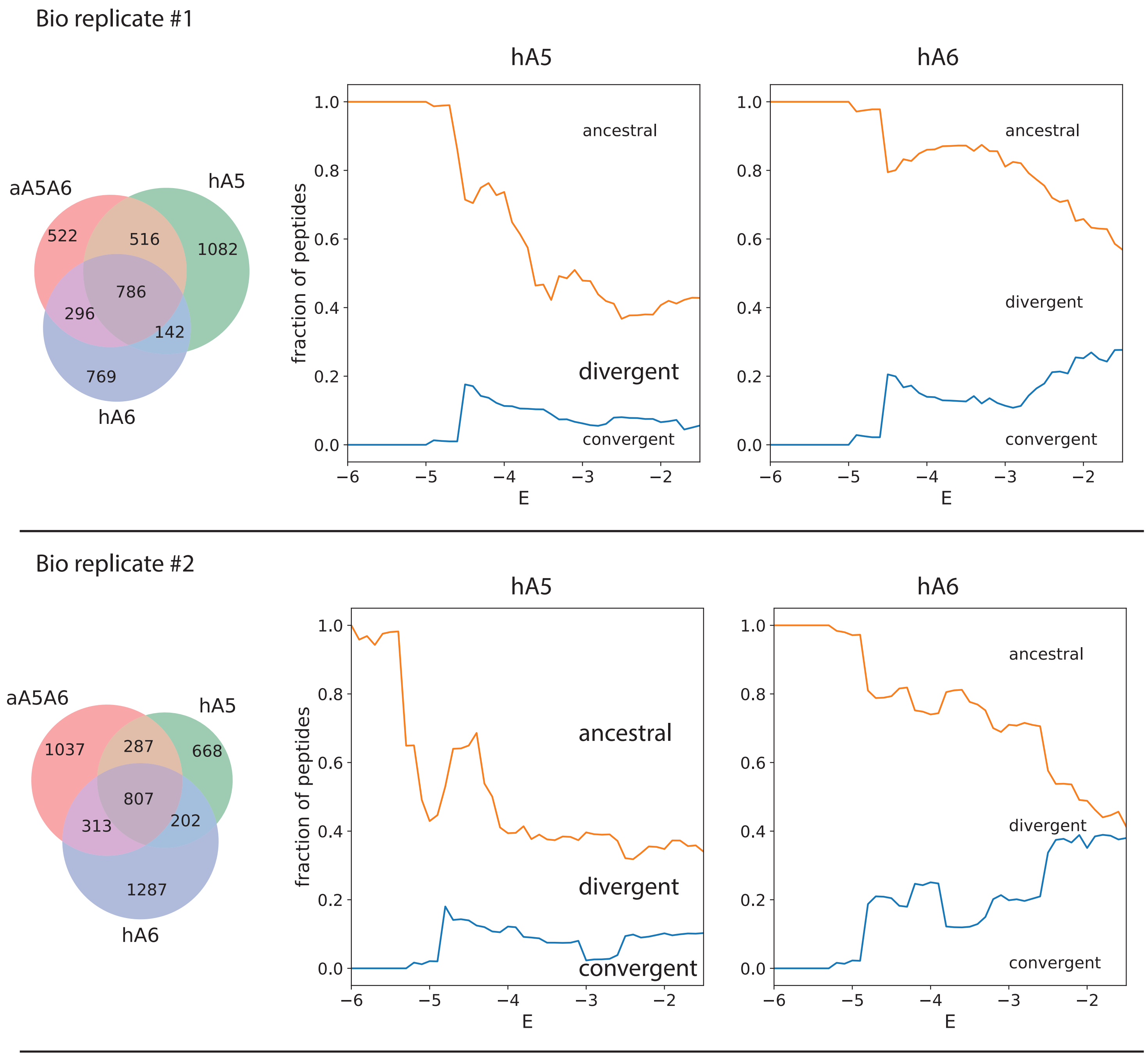

Fig S10: Main conclusion about ancestral specificity is robust to choice of bio replicate. Panels show Venn diagram calculated in panel 4A and proportions of peptides that are ancestral, divergent and convergent for hA5 and hA6 (panels 4E, F). The top row was calculated using bio-rep #1; the bottom row independently using bio-rep #2. The results are different numerically, but qualitatively the same: hA5 and hA6 evolved by acquiring new peptide targets relative to the ancestral protein.

Table S1: Number of sequencing reads for each sample. Sample, and whether or not competitor was added, are indicated on the right. Columns show biological replicates 1 or 2. “total” columns indicate reads returned by Illumina software pipeline. “good” columns indicate reads that passed our quality control and were used to calculate enrichment values.

		rep1		rep2	
sample	competitor	total	good	total	good
hA5	-	24,794,016	19,695,958	29,085,203	16,773,567
hA5	+	15,053,706	11,523,991	17,631,137	13,612,463
hA6	-	22,728,393	17,722,779	7,769,003	5,972,295
hA6	+	13,953,466	11,004,701	23,026,469	18,128,759
raw library		39,700,991	32,190,368	—	—
